# Sleep apnea and diabetes mellitus are independently associated with cardiovascular events and hospitalization for heart failure after coronary artery bypass grafting

**DOI:** 10.1038/s41598-020-78700-9

**Published:** 2020-12-10

**Authors:** Aye-Thandar Aung, Chieh-Yang Koo, Wilson W. Tam, Zhengfeng Chen, William Kristanto, Hui-Wen Sim, Pipin Kojodjojo, Theodoros Kofidis, Chi-Hang Lee

**Affiliations:** 1 Department of Cardiology, National University Heart Centre Singapore, 1E Kent Ridge Road, NUHS Tower Block Level 9, Singapore, 119228 Singapore; 2 Alice Lee Centre for Nursing Studies, National University of Singapore, Singapore, Singapore; 3 Division of Cardiology, Department of Medicine, Ng Teng Fong General Hospital, Singapore, Singapore; 4 Department of Cardiac, Thoracic and Vascular Surgery, National University Heart Centre Singapore, Singapore, Singapore; 5 Yong Loo Lin School of Medicine, National University of Singapore, Singapore, Singapore; 6 Cardiovascular Research Institute, National University of Singapore, Singapore, Singapore

**Keywords:** Interventional cardiology, Risk factors, Sleep disorders

## Abstract

The relative and combined effects of sleep apnea with diabetes mellitus (DM) on cardiovascular outcomes in patients undergoing coronary artery bypass grafting (CABG) remain unknown. In this secondary analysis of data from the SABOT study, 1007 patients were reclassified into four groups based on their sleep apnea and DM statuses, yielding 295, 218, 278, and 216 patients in the sleep apnea (+) DM (+), sleep apnea (+) DM (−), sleep apnea (−) DM (+), and sleep apnea (−) DM (−) groups, respectively. After a mean follow-up period of 2.1 years, the crude incidence of major adverse cardiac and cerebrovascular event was 18% in the sleep apnea (+) DM (+), 11% in the sleep apnea (+) DM (−), 13% in the sleep apnea (−) DM (+), and 5% in the sleep apnea (−) DM (−) groups. Using sleep apnea (−) DM (−) as the reference group, a Cox regression analysis indicated that sleep apnea (+) and DM (+) independently predicted MACCEs (adjusted hazard ratio, 3.2; 95% confidence interval, 1.7–6.2; *p* = 0.005) and hospitalization for heart failure (adjusted hazard ratio, 12.6; 95% confidence interval, 3.0–52.3; *p* < 0.001). Sleep apnea and DM have independent effects on the prognosis of patients undergoing CABG.

**Clinical trial registration**: ClinicalTrials.gov identification no. NCT02701504.

## Introduction

Diabetes mellitus (DM) is a well-established risk factor and prognostic indicator of cardiovascular outcomes. Evidence suggests that patients with DM and advanced coronary artery disease would benefit more from coronary artery bypass grafting (CABG) than from percutaneous coronary intervention^1,2^. However, data from long-term follow-up studies have revealed independent associations of DM with the occurrence of death and adverse cardiovascular events, even among patients treated with CABG^3–8^. Patients with other comorbid conditions, such as renal insufficiency and peripheral vascular disease, have particularly high cardiovascular risks^3,6^. Consequently, interest in the cardiovascular effects of concomitant comorbid conditions in patients with DM is increasing.

Sleep apnea has been identified as an important comorbid condition that is closely associated with DM^9,10^. The reported prevalence of sleep apnea in patients with DM ranged from 24 to 61%^9^. Sleep apnea is a chronic sleep disorder characterized by recurrent upper airway collapse during sleep, which leads to intermittent hypoxemia and hypercapnia. The sympathetic activation, blood pressure surges, and hypercoagulability caused by sleep apnea have been postulated as mediators of an increased cardiovascular risk^11^. Numerous studies have demonstrated the associations of sleep apnea with various short- and long-term complications after CABG, including new-onset atrial fibrillation, respiratory complications, hospital readmission, and major adverse cardiac and cerebrovascular events (MACCEs)^12–15^. These findings were corroborated by the recently published results of the SABOT (Sleep Apnea and Bypass Operation) study, which revealed an association of sleep apnea with a 1.57-fold increase in the risk of developing MACCEs^16^.

As both sleep apnea and DM are prevalent in patients undergoing CABG, it is essential to determine the prognostic effects of these concomitant disorders. In this secondary analysis of data from the SABOT study, we reported the relative and combined effects of sleep apnea and DM on the occurrence of MACCEs and hospitalization for heart failure in patients undergoing non-emergent CABG.

## Methods

### Study design

The SABOT study was a prospective, observational study that aimed to evaluate the effect of sleep apnea on the cardiovascular outcomes of patients undergoing CABG. Details regarding the methodology, patient selection, and results of the SABOT study were recently published^16^. Briefly, patients aged 18–90 years who were scheduled to undergo non-emergent CABG were invited to participate in the study. The exclusion criteria included known sleep apnea on continuous positive airway pressure therapy (patients with untreated sleep apnea were eligible for recruitment), cardiogenic shock on mechanical ventilation and/or intra-aortic balloon pump, ongoing heart failure exacerbation requiring oxygen supplementation, perceived high risk of malignant arrhythmia, long-term use of α-blocker therapy, and severe chronic pulmonary disease. Diagnoses of DM were made by a physician based on the standard criteria^17^. This study complied with the Declaration of Helsinki. The study protocol was approved by the local institutional review board (Domain Specific Review Board-C, National Healthcare Group). All participants provided written informed consent. The SABOT study has been registered with ClinicalTrials.gov (NCT02701504).

All patients who consented to participate were asked to complete the Epworth Sleepiness Scale questionnaire and the Berlin questionnaire before the overnight sleep study^18,19^. All participants underwent an in-hospital overnight sleep study using a United States Food and Drug Administration-approved wrist-worn portable device (Watch-PAT 200, Itamar Medical, Caesarea, Israel), which has been validated through in-laboratory polysomnography testing^20^. The Watch-PAT 200 measures the peripheral arterial tone (PAT), a marker of changes in arterial pulsatile volume in the finger that are regulated by α-adrenergic nerve activity in the vascular smooth muscle. This parameter reflects the sympathetic nervous system activity and was shown to be highly correlated with polysomnography data in a previous meta-analysis of 14 studies [apnea–hypopnea index (AHI), r = 0.893, 95% confidence interval: 0.857–0.920, *p* < 0.001]^20^. The Watch-PAT 200 also measures three additional channels: the heart rate (derived from the PAT signal), pulse oximetry, and actigraphy (via a built-in actigraph). Subsequently, the device uses proprietary algorithms to estimate the PAT signal amplitude, increases in heart rate and desaturation, AHI, oxygen desaturation index, and respiratory disturbance index. The participants were classified according to the presence or absence of sleep apnea, defined respectively as a Watch-PAT AHI of ≥ 15 or < 15 events per hour.

In this pre-specified secondary analysis, 1007 patients from the SABOT study were reclassified into four groups according to their sleep apnea and DM statuses. All participants were followed using a combination of clinic visits, telephone contacts, and medical record reviews. All the reported outcome events were collected and adjudicated by an independent committee that was blinded to the patients’ characteristics and sleep study results.

### Outcomes

The pre-specified primary endpoint of this study was MACCEs, defined as the four-component composite of cardiovascular mortality, non-fatal myocardial infarction, non-fatal stroke, and unplanned revascularization. The secondary endpoints included hospitalization for heart failure, all-cause mortality, and sudden cardiac death or resuscitated cardiac arrest. All endpoint events were defined according to the Standardized Data Collection for Cardiovascular Trials Initiative^21^. Clinical event data were collected by a team blinded to the sleep study results.

### Statistical analysis

We compared the difference of the demographic and clinical variables among the four groups in Tables 1, 2, 3 and 4. Categorical variables are presented as frequencies and percentages, and differences between the four groups were evaluated using the χ^2^ test. Continuous variables with normally distributed data were summarized and compared using the one-way analysis of variance (ANOVA) and are presented as means with standard deviations. Continuous variables with skewed data were compared using the Kruskal–Wallis test and are presented as medians with interquartile ranges. Kaplan–Meier cumulative incidence curves for the incidence of MACCEs, cardiovascular mortality, non-fatal myocardial infarction, non-fatal stroke, and hospitalization for heart failure were constructed and compared among the four groups using the log-rank test. The time to occurrence of MACCEs was compared between the four groups using a Cox proportional hazards regression analysis after adjusting for potential confounders. The following covariates were included in the multivariable logistic models: age, sex, body mass index, hypertension, left ventricular ejection fraction, and chronic kidney disease. Variables were chosen based on the known clinical association with adverse cardiovascular events. For variables that are highly correlated (collinearity), the most clinically relevant parameter was chosen. Backward selection method was used to determine the final model.

**Table 1:** Baseline demographic and clinical characteristics.

Characteristics	Sleep apnea (+) DM (+) (n = 295)	Sleep apnea (+) DM (─) (n = 218)	Sleep apnea (─) DM (+) (n = 278)	Sleep apnea (─) DM (─) (n = 216)	*p* value
Age, median (IQR), years	62 (57–67)	62 (56–68)	62 (56–68)	60 (56–66.75)	0.341
Male sex, n (%)	245 (83.1)	197 (90.4)	231 (83.1)	198 (91.7)	0.004
**Ethnicity, n (%)**					
Chinese	191 (64.7)	147 (67.4)	158 (56.8)	156 (72.2)	0.017
Malay	52 (17.6)	41 (18.8)	63 (22.7)	38 (17.6)	
Indian	34 (11.5)	16 (7.3)	42 (15.1)	15 (6.9)	
Others	18 (6.1)	14 (6.4)	15 (5.4)	7 (3.2)	
**Clinical measurements**					
Systolic blood pressure, mean (SD), mm Hg	126 (20)	126 (18)	127 (19)	125 (19)	0.803
Diastolic blood pressure, mean (SD), mm Hg	71 (11)	72 (10)	70 (11)	72 (11)	0.116
Body mass index, mean (SD), kg/m^2^	26.4 (4.2)	26.2 (4.4)	24.3 (3.7)	23.9 (3.4)	**< 0.001**
Neck circumference, mean (SD), cm	39.4 (3.4)	39.1 (3.5)	38.0 (3.1)	37.7 (3.0)	**< 0.001**
Waist circumference, mean (SD), cm	96.8 (10.4)	95.4 (11.7)	91.0 (9.7)	89.2 (9.2)	< 0.001
**Cardiovascular risk factors, n (%)**					
Smoking	73 (24.7)	66 (30.3)	82 (29.5)	73 (33.8)	0.028
Hyperlipidaemia	258 (87.5)	159 (72.9)	239 (86.0)	162 (75.0)	< 0.001
Hypertension	255 (86.4)	149 (68.3)	219 (78.8)	130 (60.2)	< 0.001
Insulin dependence	148 (50.1)	–	133 (47.8)	–	0.233
**Concomitant conditions, n (%)**					
Previous myocardial infarction	145 (49.2)	94 (43.1)	123 (44.2)	98 (45.4)	0.522
Previous percutaneous coronary intervention	78 (26.4)	41 (18.1)	60 (21.6)	41 (19.0)	0.118
Previous coronary artery bypass surgery	0 (0.0)	0 (0.0)	1 (0.4)	1 (0.5)	0.543
Stroke/transient ischemic attack	33 (11.2)	28 (12.8)	40 (14.4)	17 (7.9)	0.148
Chronic kidney disease	74 (25.1)	28 (12.8)	49 (17.6)	14 (6.5)	< 0.001
Chronic kidney disease on dialysis	22 (7.5)	6 (2.8)	8 (2.9)	0 (0.0)	< 0.001
Pre-existing atrial fibrillation	13 (4.4)	11 (5.0)	15 (5.4)	8 (3.7)	0.826
Pacemaker in-situ	1 (0.3)	1 (0.5)	0 (0.0)	1 (0.5)	0.745
Implantable cardioverter defibrillator in-situ	3 (1.0)	0 (0.0)	1 (0.4)	0 (0.0)	0.203

Serum estimated glomerular filtration rate (eGFR) < 60 mL/min/1.73 m^2^.

*DM* diabetes mellitus, *IQR* interquartile range, *SD* standard deviation.

**Table 2:** Sleep study and echocardiography results.

Characteristics	Sleep apnea (+) DM (+) (n = 295)	Sleep apnea (+) DM (─) (n = 218)	Sleep apnea (─) DM (+) (n = 278)	Sleep apnea (─) DM (─) (n = 216)	*p* value
**Sleep study**					
AHI, events per hour, median (IQR)	31.3 (22.0–46.1)	27.9 (19.5–43.6)	5.9 (3.2–10.4)	5.75 (2.8–10)	< 0.001
RDI, events per hour, median (IQR)	34.6 (25.2–47.9)	30.7 (23.4–47.0)	9.5 (6.2–13.9)	11.5 (7.5–15.6)	< 0.001
ODI, events per hour, median (IQR)	19.9 (12.1–36.1)	15.7 (10.2–29.8)	2.5 (1.1–5.0)	2.1 (0.7–4.5)	< 0.001
Sleep duration, hour, median (IQR)	6.5 (5.4–7.3)	6.3 (5.6–7.2)	6.3 (5.4–7.0)	6.4 (5.3–7.3)	0.501
Duration SpO2 < 90%, min, median (IQR)	5.8 (0.8–24.6)	3.7 (0.3–14.0)	0.0 (0.0–0.5)	0.0 (0.0–0.6)	< 0.001
Percentage of sleep SpO2 < 90%, %, median (IQR)	1.5 (0.2–6.3)	1.0 (0.1–3.9)	0.0 (0.0–0.1)	0.0 (0.0–0.2)	< 0.001
Epworth Sleepiness Scale, median (IQR)*	5.0 (3.0–8.0)	6.0 (3.0–9.0)	5.0 (2.0–8.0)	4.5 (2.0–7.0)	0.066
High-risk Berlin Questionnaire, n (%)*	139 (47.3)	100 (46.1)	120 (43.3)	81 (37.7)	0.157
**Transthoracic echocardiography**					
Left ventricular ejection fraction, median (IQR), %	48 (35–60)	55 (42.8–60)	55 (45–61)	55 (45–60)	< 0.001
Left ventricular ejection fraction, n (%)					< 0.001
> 50%	112 (41.3)	111 (57.2)	142 (57.5)	122 (65.9)	
30–50%	105 (38.7)	53 (27.3)	77 (31.2)	43 (23.2)	
< 30%	54 (19.9)	30 (15.5)	28 (11.3)	20 (10.8)	
Left atrium diameter, median (IQR), mm	41 (37–45)	40 (36–44)	38 (35–42)	38 (35–42)	< 0.001
Left ventricular end–diastolic internal diameter, median (IQR), mm	52 (47–56)	52 (48–57)	49 (45–53)	55 (46–55)	< 0.001
Left ventricular end–systolic internal diameter, median (IQR), mm	37 (31–45)	35 (31–42)	33 (28–39.5)	33 (29–39.5)	< 0.001
Left ventricular mass index, median (IQR), g/m^2^	109 (91–132.5)	104.5 (87.8–132.5)	99 (81.3–120.8)	96.5 (83–127)	< 0.001
Aortic root diameter, median (IQR), mm	33 (30–35)	33 (31–36)	32 (30–35)	33 (31–35)	0.003
E/A, median (IQR)	0.9 (0.7–1.4)	0.8 (0.7–1.3)	0.8 (0.7–1.2)	0.9 (0.7–1.3)	0.564
Pulmonary artery systolic pressure, median (IQR), mmHg	32 (25.6–43)	28.3 (23.5–35.2)	28 (24–34)	29 (24–35)	0.005

Four patients did not undergo the questionnaires.

*AHI* apnea–hypopnea index, *BQ* Berlin Questionnaire, *DM* diabetes mellitus, *ESS* Epworth Sleepiness Scale, *IQR* interquartile range, *ODI* oxygen desaturation index, *RDI* respiratory disturbance index, *SD* standard deviation, *SpO*_*2*_ arterial oxygen saturation.

AHI = the number of apnea or hypopnea events per hour of sleep.

RDI = the number of apnea, hypopnea, or respiratory effort related arousals per hour of sleep.

ODI = the number of times per hour of sleep that the blood oxygen level drop by at least 4% from baseline.

**Table 3:** Coronary angiography and CABG characteristics.

Characteristics	Sleep apnea (+) DM (+) (n = 295)	Sleep apnea (+) DM (─) (n = 218)	Sleep apnea (─) DM (+) (n = 278)	Sleep apnea (─) DM (─) (n = 216)	*p* value
**Clinical Presentation, n (%)**					0.138
ST–segment elevation myocardial infarction	42 (14.3)	18 (8.3)	28 (10.1)	21 (9.8)	
Non–ST–segment elevation myocardial infarction	118 (40.3)	87 (39.9)	101 (36.3)	84 (39.1)	
Unstable angina	48 (16.4)	46 (21.1)	60 (21.6)	43 (20.0)	
Stable angina	73 (24.9)	56 (25.7)	86 (30.9)	57 (26.5)	
Other	12 (4.1)	11 (5.0)	3 (1.1)	10 (4.7)	
**Number of diseased coronary vessels, n (%)**					0.216
One	10 (3.4)	10 (4.6)	5 (1.8)	9 (4.2)	
Two	28 (9.5)	28 (12.8)	34 (12.2)	34 (15.7)	
Three	257 (87.1)	180 (82.6)	239 (86.0)	173 (80.1)	
Left main artery stenosis ≥ 50%	94 (32.0)	61 (28.2)	78 (28.1)	79 (36.6)	0.161
**Operation type, n (%)**					0.234
On-pump CABG	289 (98.0)	213 (97.7)	267 (96.4)	204 (94.4)	
Off-pump CABG	6 (2.0)	4 (1.8)	10 (3.6)	11 (5.1)	
Hybrid CABG	0 (0.0)	1 (0.5)	0 (0.0)	1 (0.5)	
**Number of bypass grafts, n (%)**					0.262
1–2	71 (24.1)	48 (22.0)	50 (18.1)	50 (23.1)	
3–4	219 (74.2)	165 (75.7)	226 (81.3)	165 (76.4)	
5–6	5 (1.7)	5 (2.3)	2 (0.7)	1 (0.5)	
**Number of venous grafts, n (%)**					0.550
0–1	71 (24.1)	56 (25.7)	59 (21.1)	58 (26.9)	
2–3	220 (74.6)	158 (72.5)	217 (78.1)	157 (72.7)	
4–5	4 (1.4)	4 (1.8)	2 (0.7)	1 (0.5)	
LIMA graft, n (%)	279 (94.6)	205 (94.0)	269 (96.8)	205 (94.9)	0.497
**Non-LIMA arterial grafts, n (%)**					0.123
Radial artery or RIMA	10 (3.4)	18 (8.3)	15 (5.4)	16 (7.4)	
Radial artery and RIMA	3 (1.0)	4 (1.8)	1 (0.4)	4 (1.9)	
Concurrent valve operation, n (%)	22 (7.5)	22 (10.1)	5 (1.8)	20 (9.3)	0.001
Total operation time, minutes, median (IQR)	293 (252–329)	298.5 (261–334.5)	285 (253–318.8)	287.5 (250–329)	0.088
Estimated blood loss, mls, median (IQR)	200 (100–300)	200 (117.5–340)	200 (100–300)	200 (120–300)	0.421
**Length of stay, days, median (IQR)**					
Intensive care unit	3 (2–4)	3 (2–4)	3 (2–4)	3 (2–4)	0.087
Hospitalization	8 (6–12)	7 (6–9)	7 (6–10)	6.5 (6–9)	< 0.001

*CABG* coronary artery bypass grafting, *DM* diabetes mellitus, *IQR* interquartile range, *LIMA* left internal mammary artery, *RIMA* right internal mammary artery, *SD* standard deviation.

**Table 4:** Medications upon discharge.

Medications	Sleep apnea (+) DM (+) (n = 295)	Sleep apnea (+) DM (─) (n = 218)	Sleep apnea (─) DM (+) (n = 278)	Sleep apnea (─) DM (─) (n = 216)	*p* value
Aspirin	268 (92.4)	199 (93.0)	245 (89.4)	196 (91.2)	0.482
β-blocker	264 (91.0)	193 (90.2)	249 (90.9)	192 (89.3)	0.915
ACEI/ARB	112 (38.6)	64 (29.9)	104 (38.0)	65 (30.2)	0.063
Statin	279 (96.2)	210 (98.1)	271 (98.9)	209 (97.2)	0.190
Ezetimibe	7 (2.4)	4 (1.9)	2 (0.7)	5 (2.3)	0.438
Fibrates	7 (2.4)	5 (2.3)	7 (2.6)	1 (0.5)	0.338
**Oral anticoagulant**					
Warfarin	40 (13.8)	26 (12.1)	20 (7.3)	17 (7.9)	0.036
Direct oral anticoagulant	9 (3.1)	8 (3.7)	5 (1.8)	3 (1.4)	0.342
Frusemide	176 (60.7)	126 (58.9)	139 (50.7)	101 (47.0)	0.006
Spironolactone	23 (7.9)	17 (7.9)	8 (2.9)	10 (4.7)	0.032

*ACEI* angiotensin converting enzyme inhibitor, *ARB* angiotensin receptor blocker, *DM* diabetes mellitus.

Subsequently, the hazard ratios and 95% confidence intervals were calculated. A similar Cox proportional hazards model-based analysis was performed to compare the incidence of secondary endpoints between the four groups. The SPSS Statistics 25 software program (IBM Corp., Armonk, NY, USA) was used to calculate the descriptive statistics, obtain the Kaplan–Meier cumulative incidence curves, and perform the Cox regression analyses. All analyses were two-sided and *p* values of < 0.05 were considered to be statistically significant.

### Conference presentation

Presented at the “American Heart Association Scientific Sessions 2019”.

### Ethics approval and consent to participate

The studies were approved by the local institutional review board (Domain Specific Review Board-C, National Healthcare Group). All participants provided written informed consent.

## Results

### Baseline demographic and clinical characteristics

Between November 2013 and December 2018, 1106 patients were prospectively enrolled into the SABOT study. Of them, 1007 patients were included in this secondary analysis. First, they were reclassified into four groups based on their sleep apnea (+/−) and DM (+/−) status as follows:

1. 295 patients (29.3%) in the sleep apnea (+) DM (+) group,
2. 218 patients (21.6%) in the sleep apnea (+) DM (−) group,
3. 278 patients (27.6%) in the sleep apnea (−) DM (+) group, and
4. 216 patients (21.5%) in the sleep apnea (−) DM (−) group.

The baseline demographic and clinical characteristics of the four groups are listed in Table 1. Approximately half of the patients in each of the two DM (+) groups were receiving insulin therapy. Patients in the sleep apnea (+) DM (+) and sleep apnea (−) DM (+) groups had similar glycosylated hemoglobin levels (7.7 ± 1.5% vs. 7.7 ± 1.7%, *p* = 0.594). Patients in both DM (+) groups were more likely to be female, of non-Chinese ethnicity, and non-smokers relative to those in the DM (−) groups. Both DM (+) groups also had a higher prevalence of hyperlipidemia, hypertension, and chronic kidney disease, regardless of the sleep apnea status. Moreover, patients in both sleep apnea (+) groups had a higher body mass index than those in the sleep apnea (−) groups, regardless of the DM status.

### Sleep study results

The results of the sleep apnea screenings and sleep studies are listed by group in Table 2. As expected, patients in the two sleep apnea (+) groups had a higher AHI, oxygen desaturation index, and respiratory disturbance index and experienced a longer oxygen saturation duration of < 90% than did patients in the two sleep apnea (−) groups, regardless of the DM status. The four groups had similar scores on the Epworth Sleepiness Scale, a measure of daytime sleepiness, and similar results in the Berlin Questionnaire.

### Echocardiography

The echocardiography results are listed in Table 2. Patients in the sleep apnea (+) groups had a higher frequency of severe left ventricular dysfunction (left ventricular ejection fraction of < 30%) and a greater left ventricular mass index, left atrial diameter, and left ventricular diameter than those in the sleep apnea (−) groups, regardless of the DM status.

### Angiographic and CABG characteristics

Details of coronary angiography findings and CABG are provided in Table 3. No significant differences were observed between the four groups with regard to the indications for angiography, number of diseased coronary vessels, and involvement of the left main and proximal left anterior descending arteries. Most patients had triple vessel disease, consistent with the current indications for CABG. More than 90% of the patients underwent conventional on-pump CABG with cardiopulmonary bypass and grafting of the left internal mammary artery to the left anterior descending artery. No significant differences were observed between the four groups in terms of the CABG characteristics, number of bypass grafts, and estimated blood loss. However, both DM (−) groups were more likely to undergo a concurrent valve operation than the DM (+) groups, regardless of the sleep apnea status. Glycemic control was based on standard protocol in our intensive care unit which included hourly glucose monitoring and titration using short-acting insulin therapy until extubation. Length of stay in hospital was longer in the sleep apnea (+) DM (+) group than the other 3 groups.

### Medications upon discharge

Details about the medications used at the time of discharge are provided in Table 4. Most of the patients were using aspirin, β blockers, and statins at discharge. Patients in both sleep apnea (+) groups were more likely to use frusemide, spironolactone, and warfarin than were those in both sleep apnea (−) groups, regardless of the DM status.

### Follow-up and end points

All the study participants who underwent a sleep study were informed of the results by mail within one month. Those diagnosed to have sleep apnea were offered a referral letter to sleep clinic for evaluation. Probably these participants were overwhelmed by the CABG, post-operative recovery, and multiple clinic appointments with cardiologist and surgeon, only six patients accepted the referral and three attended the sleep clinic. None of these three patients were on CPAP therapy for more than 3 months.

**Figure 1:**
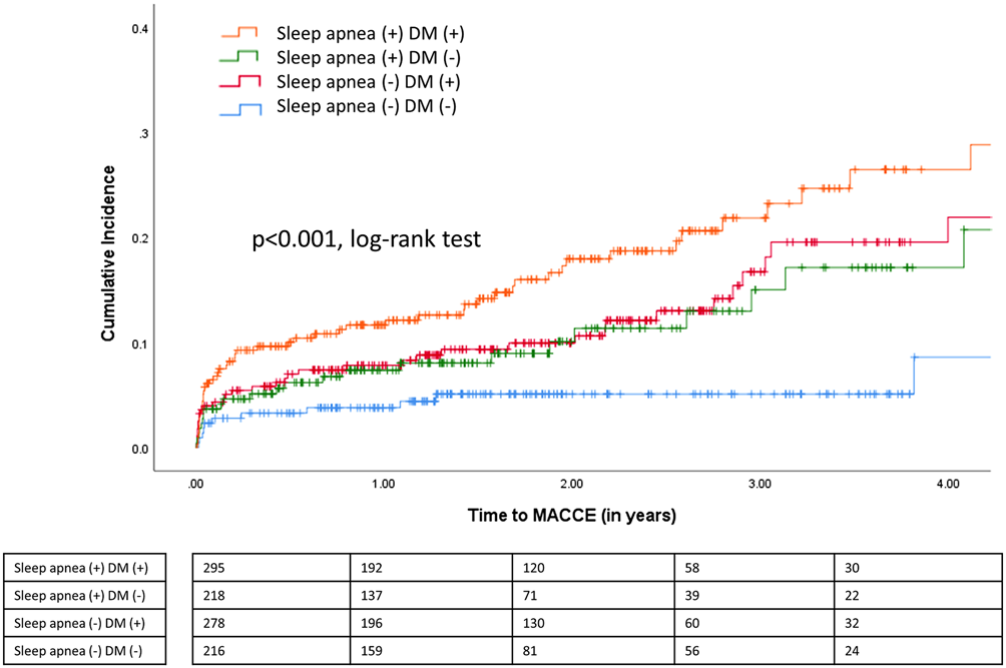
Cumulative incidence of major adverse cardiac and cerebrovascular events (MACCEs), defined as a four-component composite of cardiovascular mortality, nonfatal myocardial infarction, nonfatal stroke, and unplanned revascularization.

**Figure 2:**
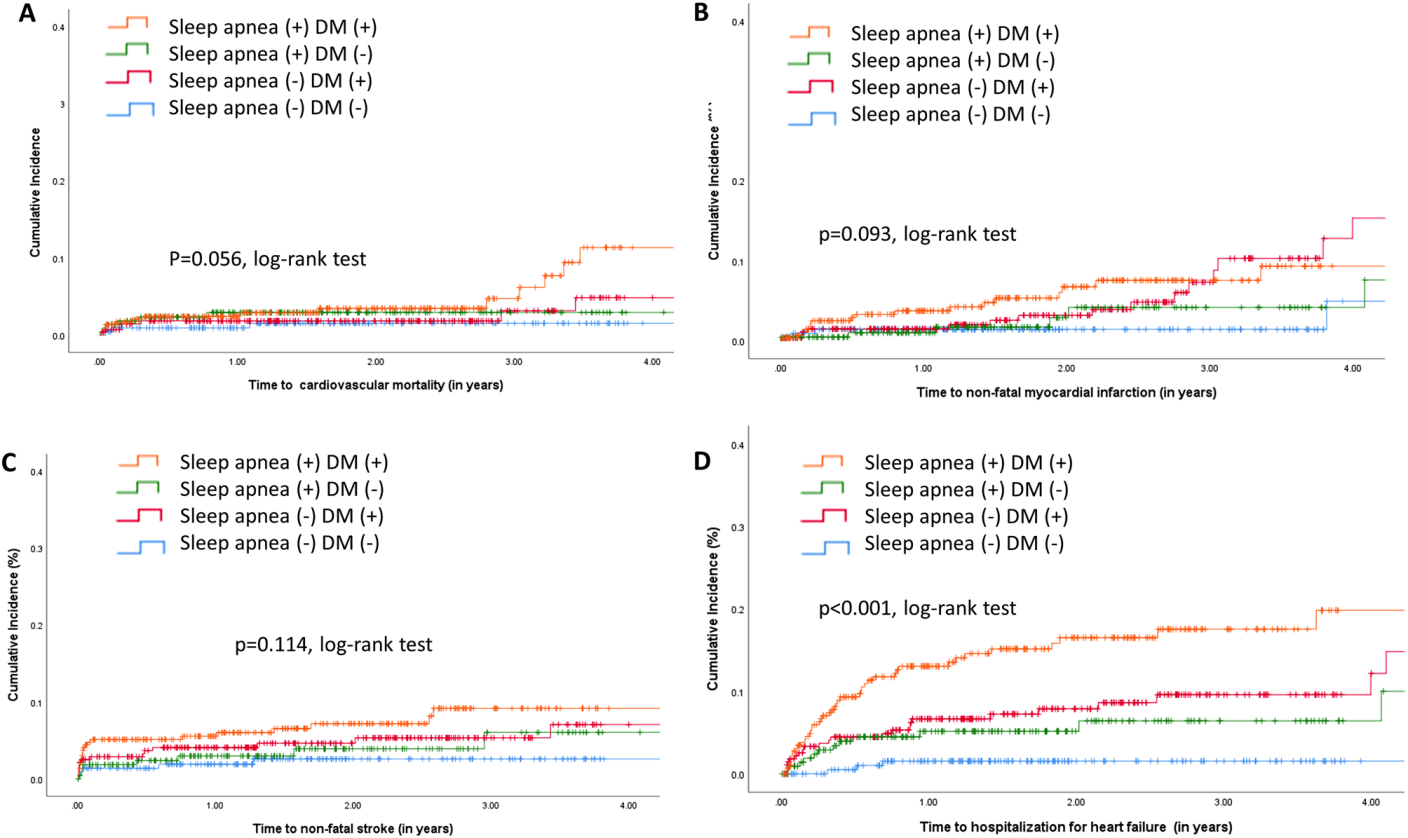
Kaplan–Meier analyses of the cumulative incidences of cardiovascular mortality (**A**), non-fatal myocardial infarction (**B**), non-fatal stroke (**C**), and hospitalization for heart failure (**D**).

Over a mean follow-up period of 2.1 years, 124 patients experienced MACCEs, including 24 cases of cardiovascular disease-related mortality, 52 cases of non-fatal myocardial infarction, and 20 cases of non-fatal stroke and 36 cases of unplanned revascularization (percutaneous coronary intervention, n = 35; repeat CABG, n = 1). The indication for unplanned revascularization was graft failure (n = 34) and disease progression of the native coronary arteries (n = 2). The crude incidence of MACCEs was highest in the sleep apnea (+) DM (+) group (18%), followed by that in the sleep apnea (−) DM (+) group (13%) and the sleep apnea (+) DM (−) group (11%), and lowest in the sleep apnea (−) DM (−) group (5%). Comparisons of the Kaplan–Meier cumulative incidence curves for MACCEs between the four groups are shown in Fig. 1. The Kaplan–Meier cumulative incidence curves for cardiovascular mortality, non-fatal myocardial infarction, non-fatal stroke, and hospitalization for heart failure are presented in Fig. 2.

The results of a multivariate Cox regression analysis of adverse cardiovascular events across the four groups are presented in Table 5. Using the sleep apnea (−) DM (−) group as the reference, the Cox regression analysis indicated that a sleep apnea (+) DM (+) status was associated with an increased risk of MACCEs (adjusted hazard ratio: 3.2, 95% confidence interval: 1.7–6.2, *p* = 0.005), including increased risks of both unplanned revascularization (adjusted hazard ratio: 3.6, 95% confidence interval: 1.0–12.5, *p* = 0.038) and hospitalization for heart failure (adjusted hazard ratio: 12.6, 95% confidence interval: 3.0–52.3, *p* < 0.001). Other independent predictors of MACCE were age (adjusted hazard ratio: 1.02, 95% confidence interval: 1.00–1.05, *p* = 0.045) and chronic kidney disease (adjusted hazard ratio: 2.1, 95% confidence interval: 1.4–3.1, *p* < 0.001). Our analysis further revealed an association of sleep apnea with MACCEs in patients with either a DM (+) status (adjusted hazard ratio: 3.2, 95% confidence interval: 1.7–6.2, *p* < 0.001) or a DM (−) status (adjusted hazard ratio: 2.1, 95% confidence interval: 1.0–4.4, *p* = 0.043, interaction *p* value: 0.473). In addition, we conducted a similar Cox-regression analysis by considering sleep apnea status, DM status and their interaction term as three independent variables, the results are summarized in Supplementary Table.

**Table 5:** Adjusted hazard ratios for adverse cardiovascular events via Cox regression.

Characteristics	Sleep apnea (+) DM (+) (n = 295)	Sleep apnea (+) DM (─) (n = 218)	Sleep apnea (─) DM (+) (n = 278)	Sleep apnea (─) DM (─) (n = 216)	*p* value
	Adjusted HR (95% CI)	Adjusted HR (95% CI)	Adjusted HR (95% CI)		
MACCE	3.2 (1.7, 6.2)**	2.1 (1.0, 4.4)*	2.4 (1.2, 4.6)*	Ref	0.005
Cardiovascular mortality	3.2 (0.9, 11.0)	1.9 (0.5, 7.4)	1.9 (0.5, 7.2)	Ref	0.250
Non-fatal myocardial infarction	2.5 (0.8, 7.6)	1.5 (0.4, 5.4)	2.4 (0.8, 7.1)	Ref	0.324
Non-fatal stroke	1.5 (0.6, 3.9)	1.0 (0.3, 2.9)	1.4 (0.5, 3.8)	Ref	0.668
Unplanned revascularization	3.6 (1.0, 12.5)*	2.2 (0.5, 8.7)	3.3 (0.9, 11.6)	Ref	0.038
All-cause mortality	2.6 (1.0, 6.8)	1.6 (0.5, 4.7)	2.0 (0.7, 5.6)	Ref	0.230
Sudden cardiac death or resuscitated cardiac arrest	3.6 (0.8, 16.2)	2.7 (0.5, 13.6)	1.9 (0.4, 9.4)	Ref	0.289
Heart failure hospitalization	12.6 (3.0, 52.3)**	4.4 (0.9, 19.9)	7.6 (1.8, 32.7)**	Ref	< 0.001
New-onset atrial fibrillation	1.0 (0.7, 1.5)	1.1 (0.7, 1.7)	0.8 (0.5, 1.2)	Ref	0.535

Age, sex, body mass index, left ventricular ejection fraction, hypertension, and chronic kidney disease were included as covariates and backward selection method was then applied.

*DM* diabetes mellitus, *MACCE* major adverse cardiac and cerebrovascular events, *CI* confidence interval, *HR* hazard ratio.

*p < 0.05; **p < 0.01.

## Discussion

In this secondary analysis of 1007 patients from the SABOT study who participated in an overnight sleep study before non-emergent CABG, we observed a high prevalence of concomitant sleep apnea and DM (29.3%). During a mean follow-up period of 2.1 years, we determined that a status of concomitant sleep apnea and DM was associated independently with a 3.2-fold increase in the risk of the four-component MACCE (cardiovascular mortality, non-fatal myocardial infarction, non-fatal stroke, and unplanned revascularization) and a 12.6-fold increase in the risk of hospitalization for heart failure. This analysis should increase awareness of the global burden of sleep apnea and DM by providing data that can facilitate the development of strategies and health policies to address this important public health problem. Our research also highlights substantial gaps in pre-CABG risk stratification, which should be very concerning because of the high prevalence and wide-ranging negative sequelae of both sleep apnea and DM.

In recent decades, the prevalence of DM has been increasing among adults worldwide. In 2017, approximately 5 million deaths among adults worldwide were attributable to DM^22^. In the same year, the total global healthcare expenditure related to DM was estimated to be USD 850 billion^22^. The prevalence of DM in Singapore is much higher than the global average (12.8% vs. 8.3%)^23^. Recently, clinicians have tended to refer patients with DM to CABG for required revascularization. These trends may explain why the prevalence of DM in the present study (56.9%) was higher than those reported in earlier studies conducted in Western countries (13–31%)^3–7^. This discrepancy and the increasing recognition of sleep apnea as an important DM comorbidity and cardiovascular risk marker inspired our secondary analysis.

Although a recent report described a bidirectional association between sleep apnea and DM^24^, the combined effects of these disorders on the cardiovascular outcomes of patients undergoing CABG have not been studied. No previous study on the association between DM and CABG outcomes has reported the additional prognostic effect of sleep apnea^3–8^. Recently, two relatively large-scale studies demonstrated an independent association of sleep apnea with adverse cardiovascular events in patients undergoing CABG and major non-cardiac surgery^16,25^. The predictive effects of sleep apnea and DM for adverse cardiovascular events were reported to be additive in patients undergoing percutaneous coronary intervention^26^. To the best of our knowledge, however, this is the first study to report a similar association in patients undergoing CABG.

Notably, a sleep apnea (+) DM (+) status seems to exert differential effects on the outcome of hospitalization for heart failure between patients undergoing percutaneous coronary intervention and those undergoing CABG. We previously reported that a sleep apnea (+) DM (+) status was independently associated with the occurrence of MACCEs, but not with hospitalization for heart failure, in patients undergoing percutaneous coronary intervention^26^. Interestingly, in our analysis of patients undergoing CABG, we observed independent associations of a sleep apnea (+) DM (+) status with both MACCEs and hospitalization for heart failure. Particularly, our secondary analysis revealed a 12.6-fold increased risk of hospitalization for heart failure after CABG. This discrepancy between our studies is intriguing but might be explained by the higher prevalence of heart failure in the sleep apnea (+) DM (+) group, which was indicated by the lower left ventricular ejection fraction on echocardiography and more frequent use of diuretic, β-blocker, and angiotensin converting enzyme inhibitor/angiotensin receptor blocker therapies in the sleep apnea (+) DM (+) group relative to the other three groups in this analysis. However, the relationship between a sleep apnea (+) DM (+) status and hospitalization for heart failure did not change after adjusting for covariates such as the left ventricular ejection fraction. As hospitalization after CABG remains a clinical challenge associated with reduced patient satisfaction and escalating healthcare costs, further studies are needed to evaluate the benefits of sleep apnea screening and treatment in patients with DM undergoing CABG, as highlighted by the International Diabetes Federation’s Task Force on Epidemiology and Prevention^27^. Furthermore, only 38% of the patients in the sleep apnea (+) DM (+) group were prescribed with angiotensin converting enzyme inhibitor/angiotensin receptor blocker upon discharge, optimization of prescription in this high-risk subgroup may be warranted.

While completeness of revascularization was not a pre-specified endpoint, it is intriguing to find that incomplete revascularization, based on percentage of patients with tripe vessel disease minus percentage of patients with at least three bypass grafts, was highest in the sleep apnea (+) DM (+) group (11.2%), followed by the sleep apnea (+) DM (−) group (4.6%), sleep apnea (−) DM (+) group (4.0%), and sleep apnea (−) DM (−) group (3.2%). This observation suggests that the high incidence of MACCE in the patients with sleep apnea (+) DM (+) group may be mediated by incomplete revascularization and residual ischemia.

This study had several limitations that should be considered. This was a secondary analysis of a completed observational study without a proper sample size calculation, which might have affected the study power for the reported endpoints. When the SABOT study was conceptualized during 2011–2012, no existing large-scale study had subjected patients to an in-laboratory polysomnography prior to CABG. As these patients faced a high level of cardiac risk, we opted for a simple wrist-worn Watch-PAT 200 sleep monitoring device to diagnose sleep apnea safely, as this device could be worn in general cardiology wards with concurrent routine nursing care. Although the Watch-PAT 200 is not considered the gold-standard diagnostic device in the sleep community, it has been subjected to validation studies^20^ and has been approved by the US Food and Drug Administration for patient use. At present, Watch-PAT 200 is not indicated for patients with atrial fibrillation. However, only less than 5% of the patients in this study have atrial fibrillation. It is unlikely that it has materially changed the study results. Information related to the diagnosis of DM was obtained from the patients’ electronic medical records. All patients underwent a pre-CABG evaluation (including fasting blood glucose and glycosylated hemoglobin monitoring) at the same hospital, which used the latest American Diabetic Association criteria to diagnose DM. However, the records did not include some details related to DM, including the disease duration, microvascular complications, or medication doses and adherence. These unknown or uncaptured confounding variables might have affected the results of this study. Moreover, follow-up coronary angiography was not conducted, and therefore, we were unable to evaluate the patency of the native coronary arteries or bypass grafts. Regarding our study population, our findings cannot be extrapolated to patients undergoing emergency CABG or major non-cardiac surgeries. Moreover, women were under-represented in our study population, which was predominantly Asian. It is uncertain whether our findings could be generalized to a Western population.

## Conclusion

In summary, we observed a high prevalence of concomitant sleep apnea and DM among patients undergoing non-emergent CABG. Patients with concomitant sleep apnea and DM had an increased risk of developing a MACCEs (cardiovascular mortality, non-fatal myocardial infarction, non-fatal stroke, or unplanned revascularization). Moreover, a status of concomitant sleep apnea and DM was independently associated with hospitalization for heart failure. The screening and treatment of sleep apnea in patients with DM who are undergoing CABG might effectively reduce the healthcare costs and negative effects associated with this condition.

## Supplementary information

Supplementary Table.

## Data Availability

The data are subject to Singapore’s personal data protection laws, and restrictions have been imposed by the Domain Specific Review Board to ensure the data privacy of the study participants. Therefore, data cannot be made freely available in a public repository. Please contact the corresponding author in case of further questions.
