# Supplementary material for: Sleep apnea and diabetes mellitus are independently associated with cardiovascular events and hospitalization for heart failure after coronary artery bypass grafting
Source: Sci Rep. 2020 Dec 10;10:21664. doi: 10.1038/s41598-020-78700-9 (PMC7730381; doi:10.1038/s41598-020-78700-9)
Supplement: Supplementary file 1 — Supplementary Table. [file 41598_2020_78700_MOESM1_ESM.docx]

**Sleep Apnea and Diabetes Mellitus Are Independently Associated with Cardiovascular Events and Hospitalization for Heart Failure after Coronary Artery Bypass Grafting**

(Running title: *OSA, DM and CABG*)

Aye-Thandar **Aung**, Chieh-Yang **Koo**, Wilson W. **Tam**, Zhengfeng **Chen**, William **Kristanto**, Hui-Wen **Sim**, Pipin **Kojodjojo**, Theodoros **Kofidis**, Chi-Hang **Lee**

**Supplementary Table. Adjusted Hazard Ratios for adverse cardiovascular events via Cox regression, considering sleep apnea status, DM status and their interaction term. CI, confidence interval; DM, diabetes mellitus; HR, hazard ratio; MACCE, major adverse cardiac and cerebrovascular events*;* and NS, not significant. Age, sex, body mass index, left ventricular ejection fraction, hypertension, and chronic kidney disease were included as covariates and backward selection method was then applied. * p<0.05, **p<0.01**

| **Characteristics** | **Sleep apnea**  **(Ref: No)** |  | **DM**  **(Ref: No)** |  | **Sleep apnea x**  **DM (Ref: No)** |
| --- | --- | --- | --- | --- | --- |
|  | **Adjusted HR (95% CI)** | **p-value** | **Adjusted HR (95% CI)** | **p-value** | **Adjusted HR (95% CI)** |
| MACCE | 1.54 (1.07, 2.22)* | 0.020 | 1.79 (1.20, 2.67)** | 0.004 | NS |
| Cardiovascular mortality | 1.72 (0.83, 3.58) | 0.147 | 1.76 (0.81, 3.83) | 0.151 | NS |
| Non-fatal myocardial infarction | 1.16 (0.63, 2.14) | 0.632 | 1.94 (0.94, 4.01) | 0.073 | NS |
| Non-fatal stroke | 1.03 (0.56, 1.90) | 0.913 | 1.52 (0.79, 2.93) | 0.216 | NS |
| Unplanned revascularization | 1.28 (0.66, 2.47) | 0.461 | 2.20 (1.04, 4.69)* | 0.040 | NS |
|  |  |  |  |  |  |
| All-cause mortality | 1.35 (0.77, 2.36) | 0.301 | 1.74 (0.96, 3.17) | 0.069 | NS |
| Sudden cardiac death or resuscitated cardiac arrest | 2.11 (0.92, 4.83) | 0.077 | 1.45 (0.63, 3.35) | 0.379 | NS |
| Heart failure hospitalization | 1.89 (1.14, 3.14)* | 0.013 | 3.67 (1.98, 6.82)** | <0.001 | NS |
| New-onset atrial fibrillation | 1.15 (0.87, 1.54) | 0.332 | 0.85 (0.64, 1.13) | 0.265 | NS |
